# THE GROWING STRENGTH OF THE ARCH NETWORK: INTERDISCIPLINARY COLLABORATIONS ACROSS INSTITUTIONS AND PRISON WALLS

**DOI:** 10.1093/geroni/igae098.1546

**Published:** 2024-12-31

**Authors:** Jennifer James

**Affiliations:** University of California San Francisco, San Francisco, California, United States

## Abstract

The ARCH network has intentionally brought together scholars from different disciplines, clinicians, lawyers, policy makers, advocates, and people with lived experience of incarceration from institutions around the globe. Too often, individuals interested in using research to advance our understanding of health and seek change in policy and practice are siloed, working independently and without the benefits of inter-professional collaboration. Importantly, most health-focused research about incarceration has been conducted at a distance, without directly engaging those who are incarcerated in the formulation of studies, analysis and interpretation, or the dissemination of findings. Given this history, members of the ARCH network conducted community-based research with people with lived experience of incarceration, their loved ones, and their healthcare providers to both identify priorities for aging research and identity barriers and facilitators to collaborating in research. We will describe how the ARCH network has created new possibilities for and understandings of collaborative research across prison walls and speak to some of the emerging research from the network. In particular, we will highlight the policy advancements, practice interventions, and community resources that have emerged from health-equity focused, collaborative, aging research on incarceration.

